# Epigenetic regulation during the differentiation of stem cells to germ cells

**DOI:** 10.18632/oncotarget.18444

**Published:** 2017-06-12

**Authors:** Yuan-Chao Sun, Yong-Yong Wang, Wei Ge, Shun-Feng Cheng, Paul W. Dyce, Wei Shen

**Affiliations:** ^1^ College of Animal Science and Technology, Institute of Reproductive Sciences, Qingdao Agricultural University, Qingdao 266109, China; ^2^ Department of Animal Sciences, Auburn University, Auburn, AL 36849, USA

**Keywords:** stem cell, germ cell, differentiation, epigenetic regulation

## Abstract

Gametogenesis is an essential process to ensure the transfer of genetic information from one generation to the next. It also provides a mechanism by which genetic evolution can take place. Although the genome of primordial germ cells (PGCs) is exactly the same with somatic cells within an organism, there are significant differences between their developments. For example, PGCs eventually undergo meiosis to become functional haploid gametes, and prior to that they undergo epigenetic imprinting which greatly alter their genetic regulation. Epigenetic imprinting of PGCs involves the erasure of DNA methylation and the reestablishment of them during sperm and oocyte formation. These processes are necessary and important during gametogenesis. Also, histone modification and X-chromosome inactivation have important roles during germ cell development. Recently, several studies have reported that functional sperm or oocytes can be derived from stem cells *in vivo* or *in vitro*. To produce functional germ cells, induction of germ cells from stem cells must recapitulate these processes similar to endogenous germ cells, such as epigenetic modifications. This review focuses on the epigenetic regulation during the process of germ cell development and discusses their importance during the differentiation from stem cells to germ cells.

## INTRODUCTION

Despite the identical genome, the development pattern and cell division are quite different between primordial germ cells (PGCs) and somatic cells. PGCs are exclusive to generate gametes, which are responsible for delivering genetic information across generations [1]. The specification of PGCs during development to become functional gametes has been shown to be regulated in part by epigenetic factors [2, 3]. Epigenetic regulation during germ cell development involves a variety of different mechanisms including DNA methylation and chromatin remodeling, as well as the interactions between them [4]. For example, global erasure of DNA methylation occurs in embryonic (E) 11.5 - E12.5 PGCs, the time at which they colonize the genital ridges in mice. This process involves drastic and temporary changes with the DNA demethylation of the entire genome being largely completed by approximately E12.5. During the global DNA demethylation of PGCs, the gene imprinting of parental origin is also erased [5]. Along with DNA demethylation, histone modifications also occur during the process of germ cell development. Immunostaining experiments revealed that during the specification of PGCs dynamic and orderly histone modifications take place concurrent with DNA demethylation [6]. Specifically, heterochromatin marks H3K9me2, H3K9me3 and H3K64me3 are transiently lost from PGCs at E8, E11.5 and E12.5, respectively [7, 8]. Many reports have found that epigenetic regulations play important roles in germ cell specification and repression or activation of germline special genes, which are necessary for the proper differentiation into functional gametes. These epigenetic modifications regulate the expression of germ cell special genes and guarantee the normal development of genetic information transmit across generations.

Many studies have differentiated stem cells into PGC-like cells (PGCLCs) and gamete-like cells contributing to a better understanding of mechanisms during germ cell development [9–15]. Some have also shown that epigenetic regulation plays an important and necessary role in the formation of functional gametes. But up to now, functional gametes and healthy progeny only can be induced from embryonic stem cells (ESCs) and induced pluripotent stem cells (iPSCs) - derived germ cells [9, 10]. Germ cells that derived from many other stem cells fail to give rise to functional haploid or live offspring. Improper or deficiency epigenetic modification may be one of the underlying reasons. In this review, we will discuss the importance of epigenetic regulation in germ cell development including germ cell differentiation from stem cells.

## EPIGENETIC REGULATION DURING THE DEVELOPMENT OF ENDOGENOUS GERM CELLS

**Figure 1:**
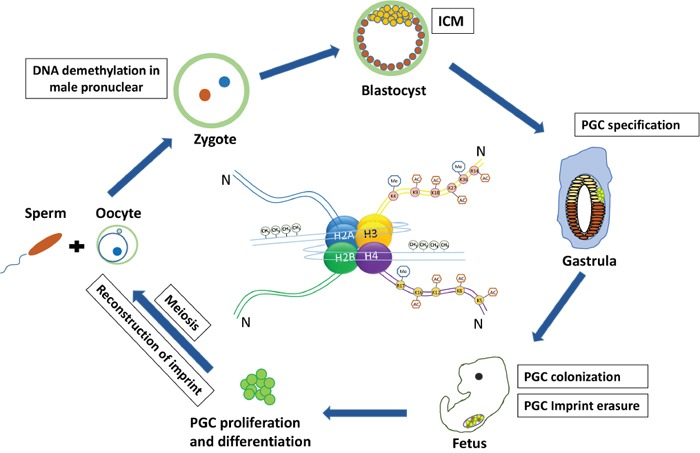
The germ cell cycle in mouse and associated epigenetic regulation Sperm and oocyte merge to form a single zygote with two pronuclei, within a short time, the male pronucleus undergoes erasure of DNA imprint methylation. After primordial germ cell (PGC) specification they migrate and colonize the genital gonad and at this time PGCs experience drastic DNA demethylation and imprint erasure. Then PGCs enter meiosis successively and reconstruct the imprints to form functional gametes.

Different to the other cell types, germline cells are unique and important because of the capability to give birth to a new individual. Because of their immortal nature and special responsibility, it is of great importance for germ cells to proceed proper proliferation of mitosis to acquire a certain quantity, and then take on meiosis to form the final functional gametes under the regulation of multiple mechanisms. Along with transcriptional regulation and relevant signaling regulatory networks, it has been reported that epigenetic regulation also plays an important role in the formation of functional gametes (Figure 1).

### Epigenetic regulation during PGC fate commitment

PGCs, the precursors of both sperm and oocyte, first arise from the extra-embryonic mesoderm at E7.25 in mice [16, 17]. When it comes to the precursors of PGCs, it is well accepted that they are induced from epiblast cells under the regulatory signals from neighbor ectoderm cells [18–22]. BMP signal and BLIMP1 (PR-domain-containing 1) are reported to be necessary and important in the specification of PGCs from epiblast cells [23]. Nevertheless, the underlying mechanisms of these processes remain largely unknown. A good perception about the precise mechanisms during this process, as well as the role of epigenetic modifications in both germ cells and somatic cells, will be helpful for us to dispose this issue. Leading to the specification, the PGC precursors receive induced signals and suppress the somatic gene expression. Besides, the formed PGCs are found to experience drastic epigenetic regulations, such as DNA demethylation at genome-wide, imprints erasure of parental, and histone modifications [24–27]. In *Drosophila* germline, PGC precursors were found to increase the levels of histone H3 lysine 9 methylation (H3K9me) but reduce levels of H3K4me, these epigenetic regulations were shown to involve in the repress of the somatic programme [28, 29]. According to the above results, we can infer that these epigenetic regulations may serve as a control switch to decide the active or repressive status of germ line in *Drosophila*.

In mice, it was reported that Prdm1-positive PGC precursors at around E6.5 have genome-wide epigenetic modifications, such as trimethylation of H3K4 and acetylation of H3K9, apparently indistinguishable from their somatic neighbors [25]. At about E7.5-E8.5, mouse PGCs begin to migrate from outside of the embryo and arrive at the undifferentiated genital ridge after specification [30, 31], during which, status of epigenetic regulations in PGCs exhibit obvious changes. During the migration, about E8.5 in mice, PGCs seem to be a progressive cell-by-cell process, with nearly all the PGCs exhibiting low H3K9me2 level by E8.5 [32, 33]. When they arrive at the genital ridge at E10.5-12.5, PGCs take place drastic epigenetic reprogramming, showing DNA demethylation and erasure of parental imprints. The DNA demethylation or erasure of imprinted genes persists until new imprints are rebuilt during gametogenesis [34]. The TET family genes are reported to play important roles in PGCs genome-wide DNA methylation. For example, TET1, whose function is to remove aberrant stochastic DNA methylation from high or intermediate CpG promoters (HCPs and ICPs), may convert 5mC into 5hmC following passive demethylation, which consequently regulate DNA methylation fidelity in PGCs [35]. Meanwhile, chromatin changes happen along with the reprogramming. On the whole, a rapid loss of histone H1 and concomitant loss of H3K9me3 and H3K27me3, as well as the prominent chromatin changes showing here are intimately linked with the process of genome-wide DNA demethylation.

Meiosis is a unique process of germ cells in many multi-cellular organisms. Meiotic events are regulated by epigenetic programs including DNA modifications and chromatin remodeling. Also, quite a number of factors that in connection with histone modification are essential for the initiation of meiosis, such as H3K9me2, whose levels keep at a low status in mitotic PGCs or spermatogonia, but exhibit dynamically changes upon the initiation of meiosis [32, 33]. When it comes to the leptotene phase of meiosis, histone variant, H2AX, is phosphorylated at γH2AX, whose accumulation is known to play an important role in triggering meiotic sex-chromosome inactivation (MSCI) and govern the DNA repair to damaged chromatin [36]. Research showed that γH2AX disappears subsequently from autosomes before pachytene when synapsis of homologous chromosomes is completed [37]. Gene-knockout studies clearly demonstrated the function of histone modifications during the process of meiosis. It was reported that Suv39h2 the H3K9 double mutations germ cells showed abnormal meiotic status in the mammalian testis. Further investigation found that the mutant spermatocytes exhibit low levels of H3K9me3 and these cells arrest at the pachytene stage of meiosis, even leading to obvious apoptosis [38]. PR domain-containing 9 (PRDM9) is one of the H3K4 methyltransferase that is specifically expressed in early meiotic germ cells. Prdm9 knock out in mice proved that Prdm9 is necessary for chromosomes synapsis of homologous during meiotic prophase, and deficient of Prdm9 repressed the expression of autosomal genes in spermatocytes [39]. These results suggest a functional link between H3K4 methylation and meiosis-specific gene expression. In addition, histone modification enzymes that with opposing functions are proved to be necessary for meiosis process. For example, mutations in enzymes that generate the repressive H3K9 methylation and the active H3K4 methylation both exhibit failure in meiotic process during spermatogenesis in mice [40, 41]. All of these reports demonstrated that early germ cell development involve multitudinous epigenetic regulations, and these regulations might work together in contributing PGCs specification, PGCs epigenetic reprogram and onset of meiosis.

### Epigenetic regulation during gametogenesis

In mice, after colonization in the fetal gonadal tissues, PGCs initiate the developmental programs to form sperms or oocytes. In the female, mitosis active PGCs expand their numbers in fetal mouse ovaries and enter into meiotic prophase at about E12.5, then most of them arrest at meiotic prophase I (MPI) at about E17.5 to birth [42–44]. In the male mouse, PGCs or the following spermatogonium would not enter into meiosis until one week after birth [45]. During the gametogenesis, rebuilding the imprinting in the male and female germline is unavoidable and necessary. This reestablishment initiates next following the sex determination, and development of gonocytes diverges to give rise to sperms or oocytes. In the male mouse, paternal methylation imprints, are progressively added to prospermatogonia from E14.5 to birth [46]. DNMT3A, the *de novo* DNA methyltransferase, was reported to have an important role in the methylation of all known paternally methylated imprints. Whereas, DNMT3B, is involved only at the Rasgrf1 locus. Except DNMT3A and DNMT3B, this establishment process needs another methyltransferase, DNMT3L, which possess no methyltransferase activity but stimulates DNMT3A and/or DNMT3B activities [47–49]. In the female mouse, reestablishment of maternal methylation imprints occurs after birth, along with the oocyte growth. The *de novo* methylation process would not complete until the fully-grown oocyte stage. Different to male germ cells, both DNMT3A and DNMT3L have essential roles in this process, but DNMT3B seems dispensable [50, 51].

After two meiotic divisions, the gonocytes undergo final developmental diverges to form mature sperms or oocytes and allow them to possess the ability of fertilization. This process of gamete maturation also involve abundant epigenetic changes. During the maturation of sperm, remarkable global chromatin remodeling occurs, leading nuclei compression of male germ cells. One of the major processes is the replacement of nucleosomal histones by protamines (PRMs) in mammalian. PRMs are small proteins which exhibit arginine-rich characteristic and are evolutionarily related to histone H1. These properties endow them the ability to package paternal DNA more efficient, which is very important in the maturation and downsizing of sperm, providing a safe environment for the genome, resistant to physical damage and chemical agents [52]. During this process, the histone variants of testis-specific linker, H1T2 and HILS1, occurs at this period and play important roles in DNA package during spermiogenesis [53]. Although protamine replacement happens in most of the sperm genome, many regions retain nucleosomal histones, which are enriched in specific epigenetic modifications, such as trimethylated H3K4, trimethylated H3K27, and unmethylated DNA.

In oocyte, throughout the process of meiosis, few changes were found in histone modifications. After the first meiotic division, the ovulated MII oocyte is stimulated by a sperm to complete the second meiotic division and achieve fertilization to form a zygote. For the zygote, oocyte contributes not only the most nutrition, maternal genome, but also associated epigenetic information and factors that are required for post-fertilization reprogramming. Despite the fundamental nature of this process, the clear molecular mechanisms of epigenetic modifications involved in late germ cell development remains largely unknown, and more work need to be done to achieve the underlying truth.

## EPIGENETIC REGULATION DURING GERM CELL DIFFERENTIATION FROM STEM CELLS

Recent studies have reported that stem cells possess the potential and ability to differentiate into germ cells [54–60]. In the year of 2011 and 2012, functional gametes including sperm and oocyte were derived from mouse ESCs and iPSCs [9, 10]. Among the numerous studies demonstrating germ cell differentiation from stem cells, limited studies have examined the epigenetic regulation between *in vitro* derived germ cells and endogenous germline cells.

### Stem cells derived PGCLCs display resemble DNA methylation with endogenous PGCs

In most germ cell induction research, differentiating stem cells to PGC phase is a necessary and important course, because it provides the differentiation more possible to success and more inspires to explore the mechanism of germ cell development. Thus, how to induce and identify the stem cells differentiated PGCLCs is of great importance. Although the gene expression profile of PGCs is quite different from somatic cells, many genes, such as *Oct-4*, *Nanog*, whose expression are found in both PGCs and ESCs in mammalian. The resemblance between gene expression profiles of PGCs and ESCs makes it hard to identify and isolate PGCLCs that are derived from differentiating ESCs. One possibility is to identify and analyze the PGCLCs by comparing their epigenetic modifications to that of ESCs. It was reported that the differentiated PGCLCs from porcine skin-derived stem cells (pSDSCs) showed a resemble methylation status compared to endogenous PGCs when treated by sodium bisulfite and sequenced to identify the methylation status of imprinted gene H19 in differentiated PGCLCs [11]. Detailedly, about 20 % of the CpG sites kept methylated in the pSDSCs, and this epigenetic status was similar to the undifferentiated fetal skin cells. Whereas at day 25 of differentiation, the PGCLCs that induced from pSDSCs, displayed highly unmethylated profile in DMR1 (99 % of CpG sites), which was very similar to porcine oocytes having 98 % of the CpG sites unmethylated [11]. Although, H19 methylation status of porcine endogenous PGCs were not present directly in that study, an epigenetic reprogram profile or demethylation occurred during the differentiation from porcine SDSCs to PGCLCs. A similar result was demonstrated in the induction from mouse ESCs or iPSCs to functional male germ cells. In this research, the methylation states of imprint genes *Igf2r*, *H19*, *Snrpn*, and *Kcnq1ot1* in PGCLCs were analyzed and compared to undifferentiated ESCs. It showed that the PGCLCs had a reduced level of methylation at CpG sits of *H19* and *Kcnq1ot1*, suggesting an imprint erasure profile in these induced PGCLCs [9]. Another study differentiated spermatogonial stem cells (SSCs) to oocytes directly, and found that although the maternally imprinted *Snrpn* kept methylated, the imprint genes *H19* and *Dik-Gtl2/meg3* were highly unmethylated in the derived-oocytes compared to SSCs [61]. These results suggest that the epigenetic reprogram or imprint erasure present with the differentiation from stem cells to germ cells, may be consistent with the DNA methylation pattern of endogenous PGCs.

### Chromatin remodeling during germ cell specification from stem cells

Except DNA methylation, histone modifications or chromatin remodeling were also found during the differentiation of germ cells from stem cells. The mentioned study found that PGCLCs differentiated from ESCs and iPSCs showed an elevated level of H3K27me3 but a reduced level of H3K9me2 and 5mC compared to endogenous PGCs [9]. Further study confirmed that levels of H3K9me2, H3K27me3 and 5mC changed drastically at different period of the whole differentiation. Firstly, from ESCs to epiblast-like cells (EpiLCs), levels of H3K9me2 and 5mC increased but decreased significantly in the induction from EpiLCs to PGCLCs. Interestingly, the level of H3K27me3 showed an opposite status, it decreased during ESCs to EpiLCs but in return increased during the differentiation from EpiLCs to PGCLCs. These complex but regular changes of 5mC levels and histone modifications during PGCLC formation are in accord with the development of endogenous PGCs. Mechanisms and functions underlying these dynamic changes of histone modifications need to be revealed. One of the opinion is that expression of germline genes may be controlled by histone modifications or DNA demethylation. To our knowledge, PGCs would upregulate a series of germline genes to maintain the undifferentiated state, following progress into meiosis and repress transposons [62, 63]. It remains unknown how histone modifications regulate the expression of these germline genes during early PGC development. One study in 2015 explored the epigenetic regulation about chromatin remodeling associated with germline gene expression during the PGCLC induction *in vitro*, and find that most of histone modifications were kept in a repressed status [64]. In their study, *Ddx4* and *Dazl*, the very important and specific genes for the development of germ cells, showed a deficiency of H3K4me3 and H3K27ac during the whole PGCLC induction, in spite of a low-level expression at day 6 of PGCLC differentiation *in vitro*. Interestingly, a high level of H3K27me3 and H3K9me2 were present in most of the germline genes during this induction because of the lack of H3K4me3, although level of H3K4me2 decreased at day 6 of PGCLC induction. These results are in accordance with the published data about natural PGC development, which elucidating the level of H3K27me3 and its influence on expression of germline genes in E11.5 PGCs. In summary, there may be a negative correlation between level of H3K27me3 and expression of germline genes, so it was proposed that, just unlike somatic cells, expression of the germline genes are suppressed or rather regulated by special histone modifications during the specification of PGCs and PGCLCs as well as the following development.

### Epigenetic regulation in the stem cell-derived gametes

Gametes produced from stem cells are generally tested for function via *in vitro* fertilization (IVF), therefore the epigenetic status of them may remain largely unexplored. Compared to the internal environment, gametes induced from stem cells *in vitro* or partial *in vivo*, lacking the normal conditions and necessary epigenetic regulations would make themselves dysfunctional. In yeast, mutations of H4S1A, which is a member of the Ste20 kinase family, will lead to defective sporulation [65]. In mice, demethylation of H3K9me2/1 plays an important role in increasing the expression of both *Tnps* and *Prms*, which are important to the development of sperm as mentioned. Besides, HDM1A/2A (the JmjC-domain-containing histone demethylase 1A/2A) knockdown male mice turn out to be infertile and have smaller testes compared to normal mice, because of dysfunction in methylation of H3K9 and acetylation of H3/H4, which is crucial for histone replacement and chromosome condensation [66, 67]. In 2011, Hayashi *et al*. successfully obtained functional sperm induced from PGCLCs by testis injection to the recipient mice. Following fertilization with oocytes by intracytoplasmic sperm injection (ICSI), the acquired zygotes gave rise to totally healthy offsprings, which have functional placentas and normal imprinting patterns. However, another research to obtain functional oocytes from PGCLCs in 2012, they examined the zygotes formed by PGCLCs, and found that nearly half of these formed zygotes had three pronuclei (3PN) [10]. While most (about 86 %) of zygotes obtained from endogenous PGC showed a normal two pronuclei (2PN) [9]. To confirm this, they scrutinized zygotes derived from H14 ESC-derived PGCLCs, and found that these zygotes also bearing 3PN at a high percentage, which is resemble to the mentioned PGCLCs-derived zygotes. Further analysis demonstrated that the most 3PN zygotes had one paternal chromosome but two maternal chromosomes, which is positive for H3K9me3. There's no doubt that all of the 3PN zygotes cannot obtain healthy offsprings, because of failure in extruding the second PB and form a triploid phenotype, leading to a low percentage of baby birth from the oocytes induced from PGCLCs. Consistent with these findings, normal gene expression and epigenetic regulations including chromatin remodeling are necessary and crucial for both PGC and PGCLC specification, as well as the function of induced gametes.

## CONCLUSIONS AND PERSPECTIVES

**Figure 2:**
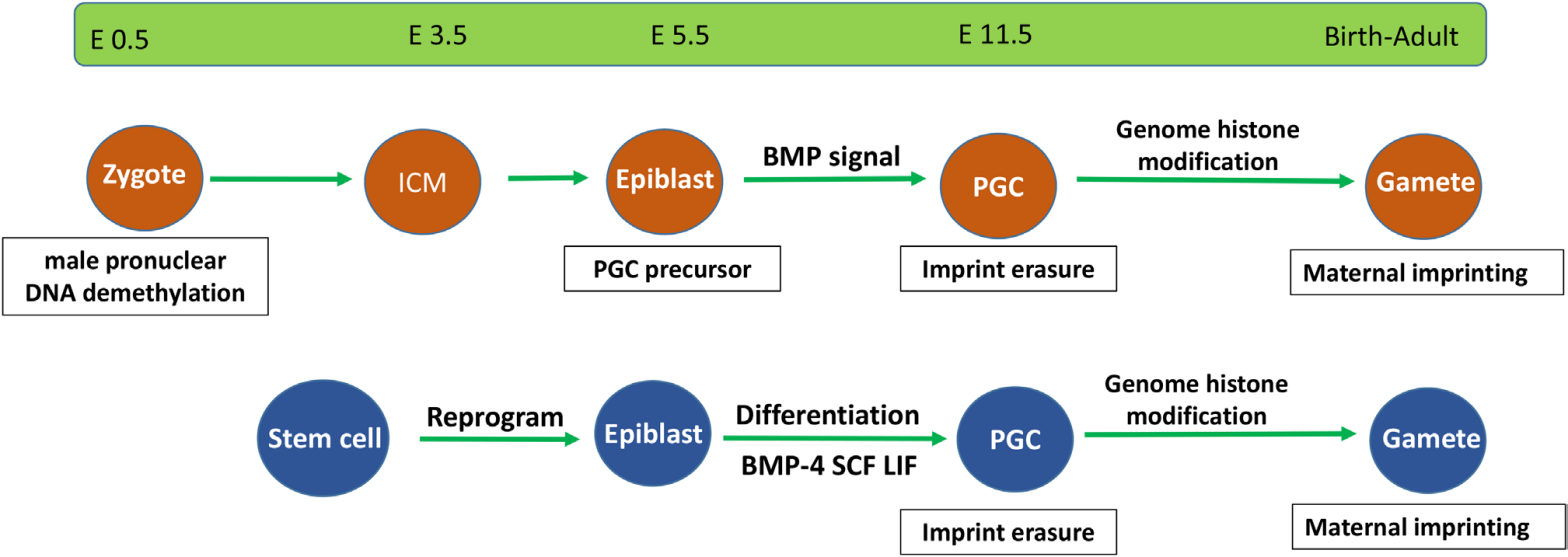
Epigenetic events in endogenous and stem cell-derived germ cells Stem cell-derived germ cells require similar development processes as those of endogenous germ cells. They both undergo epiblast differentiation and experience DNA demethylation and imprint erasure during PGC formation.

In recent years, plenty of studies have revealed that many epigenetic modifications, including DNA methylation and histone modification, have important roles in the development of germline cells. These epigenetic regulations function throughout the whole cycle of germ cell fate, including PGC specification and DNA demethylation and erasure of parental imprints, and germ cell meiotic entry, as well as the formation and maturation of functional gametes. Recent years, a series of studies have reported that stem cells possess the potential to form germ cells, which is meaningful in treatment of infertility [54–60]. Just like endogenous germline cells, the specification and following development of induced germ cells from stem cells, would also regulated by epigenetic modifications. In addition, the stem cell-derived PGCLCs show a similar epigenetic profile to the endogenous PGCs (Figure 2), and although the gametes obtained from PGCLCs can give rise to healthy offspring, some of them would fail because of abnormal karyotype. However, it remains difficult to investigate epigenetic regulation at the level of target genes at a specific stage in germ cells. Furthermore, the mutual links between DNA methylation and histone modification also need further investigation. New technology or mentality that attributed to genetic and epigenetic modifications, which can significantly increase the efficiency and quality of induced germ cells, will be of great helpful not only for the treatment of sterility infertility, but also for enriching our knowledge about function of epigenetic regulations during germ cell development and induction of germ cells from stem cells.
